# Ranking near-native candidate protein structures via random forest classification

**DOI:** 10.1186/s12859-019-3257-8

**Published:** 2019-12-24

**Authors:** Hongjie Wu, Hongmei Huang, Weizhong Lu, Qiming Fu, Yijie Ding, Jing Qiu, Haiou Li

**Affiliations:** 1 School of Electronic and Information Engineering, Suzhou University of Science and Technology, Suzhou, 215009 China; 2 Jiangsu Province Key Laboratory of Intelligent Building Energy Efficiency, Suzhou University of Science and Technology, Suzhou, 215009 China

**Keywords:** Protein structural prediction, Random forest, SPICKER

## Abstract

**Background:**

In ab initio protein-structure predictions, a large set of structural decoys are often generated, with the requirement to select best five or three candidates from the decoys. The clustered central structures with the most number of neighbors are frequently regarded as the near-native protein structures with the lowest free energy; however, limitations in clustering methods and three-dimensional structural-distance assessments make identifying exact order of the best five or three near-native candidate structures difficult.

**Results:**

To address this issue, we propose a method that re-ranks the candidate structures via random forest classification using intra- and inter-cluster features from the results of the clustering. Comparative analysis indicated that our method was better able to identify the order of the candidate structures as comparing with current methods SPICKR, Calibur, and Durandal. The results confirmed that the identification of the first model were closer to the native structure in 12 of 43 cases versus four for SPICKER, and the same as the native structure in up to 27 of 43 cases versus 14 for Calibur and up to eight of 43 cases versus two for Durandal.

**Conclusions:**

In this study, we presented an improved method based on random forest classification to transform the problem of re-ranking the candidate structures by an binary classification. Our results indicate that this method is a powerful method for the problem and the effect of this method is better than other methods.

## Background

Proteins are basic elements involved in biological functions. Recent advances in computational methods and algorithmic efficiency have enabled prediction of the three-dimensional (3D) structures of proteins from their sequences, which represents an increasingly important method for exploring their roles, networks, functions, and potentials as drug targets. Whether comparative modeling, protein threading modeling, or ab initio modeling, detecting the lowest free energy model (best model) from decoys by clustering represents an important step in protein-structure prediction [1]. In these methods, decoys are clustered, and the centroid structures of each cluster are reported as the final predicted structures. In popular protein-structure-prediction systems, including I-TASSER [2], MODELLER [3], and Rosseta [4], clusters are created iteratively. One criterion for clustering involves choosing decoys with more neighbors over decoys with fewer neighbors. The cluster centers ranked according to cluster size and suggested that larger cluster centers are closer to the best near-native models.

Zhang and et al. [5] developed SPICKER, which uses a simple and effective strategy to identify near-native conformations via cluster analysis. In the strategy, the best of the top five identified folds has a root-mean-square deviation (RMSD) from the native structure in the top 1.4% of all decoys. For 78% of the proteins, the difference in the model RMSD from the native structure and that of the native structure to the absolutely best individual decoy is < 1 Å. Li and Ng [6] proposed Calibur, which uses three strategies to enhance performance, which remains stable, regardless of increases in the number of decoys, and Francois et al. [7] proposed a fast method effective for large-scale models. Clusco [8] was developed to compare high-throughput protein models using different similarity measures, including those generated using parallel execution on CPUs and GPUs. Li et al. [9] proposed an efficient clustering method allowing rapid estimation of cluster centroids and efficient pruning of rotation spaces. Although these methods improved the accurate detection of optimal near-native models and accelerated the clustering process, their accuracy is lacking, as usually cluster centers harboring the largest models might include the closest model to the native structure due to inaccuracies related to evaluating the lowest free energy and 3D distance metrics. These stat-of-art methods have successfully explored the best five or three candidate structures from the decoys, but unfortunately sometime they failed to give a correct order of the five or three candidate structures. The accuracies of SPICKER, Calibur, and Durandal in predicting the first model are 60, 44, and 49%, respectively, with 17, 31, and 27 incorrectly ranked models in candidates, respectively. If we can re-rank the candidate structures in 100% correct order, the average RMSD of the first model can be improved 11.9, 16.3 and 15.9% with SPICKR, Calibur, and Durandal.

To address this issue, we propose an algorithm based on random forest classification to re-rank candidate structures detected by clustering. The algorithm solves the problem of re-ranking candidate structures by an binary classification, taking the length of the protein, PSSM (position-specific scoring matrix), the size of each cluster category associated with the protein, the average RMSD and average TM_SCORE [10] between the models and the other four models, and the average RMSD and average TM_SCORE between each model and all other models in the cluster category as features. Finally, the RMSD between each protein and its corresponding native protein is used as a label. Our results suggest that the algorithm chooses the first models were closer to the native structure in 12 of 43 cases versus four for SPICKER, and the same as the native structure in up to 27 of 43 cases versus 14 for Calibur and up to eight of 43 cases versus two for Durandal.

## Method

### Cluster methods for detecting candidate near-native structures

Protein-structure clustering is an important step in protein 3D structure, function, and interaction predictions. Structure-prediction methodologies involving clustering require identification candidate structures with the highest degree of similarity to the native structure from a large number of decoy structures, generated by the free modeling or template modeling, based on 3D structures similar to those provided to the clustering algorithm. The following three methods represent current methods for detecting near-native models.

#### SPICKER

The method developed by Zhang and et al. [5] generates clusters in a single-step process using a set of shrinking scales, followed by dynamic adjustment of the conformational-similarity threshold between candidate pairs during each iteration. After labeling a set of 1489 non-homologous proteins representing all protein structures in the PDB > 200 residues, a fast algorithm for population-based protein structural model analysis was proposed. Two new distance matrices for describing the differences and similarities among models were developed. Compared with existing methods using calculation times quadratic to the number of models, Dscore1-based clustering achieves linear-time complexity to obtain almost the same accuracy for near-native model selection.

#### Calibur

The method developed by Li and Ng [6] clusters decoys using proximate decoy organization, preliminary screening via lower and upper bounds, and outlier filtering. This method scales well with respect to increases in the number of decoys and automatically discovers a suitable threshold distance for clustering based on the decoys used as input. Several algorithms for this discovery are implemented in Calibur, with the fastest used by default.

#### Durandal

The method developed by Francois and et al. [7] works on large decoy sets and is consistently faster than other methods in the performance of exact clustering. In some cases, Durandal also outperforms approximate methods, with this attributed to its use of triangular inequality to accelerate exact clustering without compromising the distance function.

Although these three clustering methods can detect near-native models, the limitations of clustering methods and three-dimensional structure-distance evaluation make it difficult to determine the exact order of the candidate structures. Therefore, we chose to use random forest classification to re-rank the near-native models obtained by the three clustering algorithms.

### Inter-cluster and intra-cluster features

Feature selection is one of the key issues of the any machine learning method. The complex biological evolutionary process increases the difficulty of feature selection [11, 12]. This re-rank task is closely related to the protein and the cluster information, so we divided the seven features employed by the method into three categories: protein features, intra-cluster features (information within each cluster) and inter-cluster features (relationships between clusters). Proteins features are directly related to the protein information include 1) the length of the protein sequence and 2) position-specific scoring matrix, PSSM which is a way of encoding amino acids. The type of the PPSM is a matrix which has N lines that represent the number of amino acid in the protein and M columns that the number of types of amino acid. We converted this matrix into an vector of length 1 × (MAX*N* × *M*) and spliced it into a vector of length 6 + MAXN × *M* with the other six features. If N is greater than *MAXN*,we take *MAXN*. Intra-cluster features include the following: 3) the size of the clusters, which means the number of elements in the clusters; 4) the average RMSD between the cluster center and the remaining models in the cluster which represents the similarity of intra_cluster; and 5) the average TM_SCORE between the cluster center and the remaining models in the cluster which represents the similarity of intra_cluster. Inter-cluster features include the following: 6) the average RMSD between the current center model and the other four center models, which represents the similarity of inter_cluster; 7) the average TM_SCORE between the current center model and the other four center models, which represents the similarity of inter_cluster.

### The schematic of the method

**Fig. 1:**
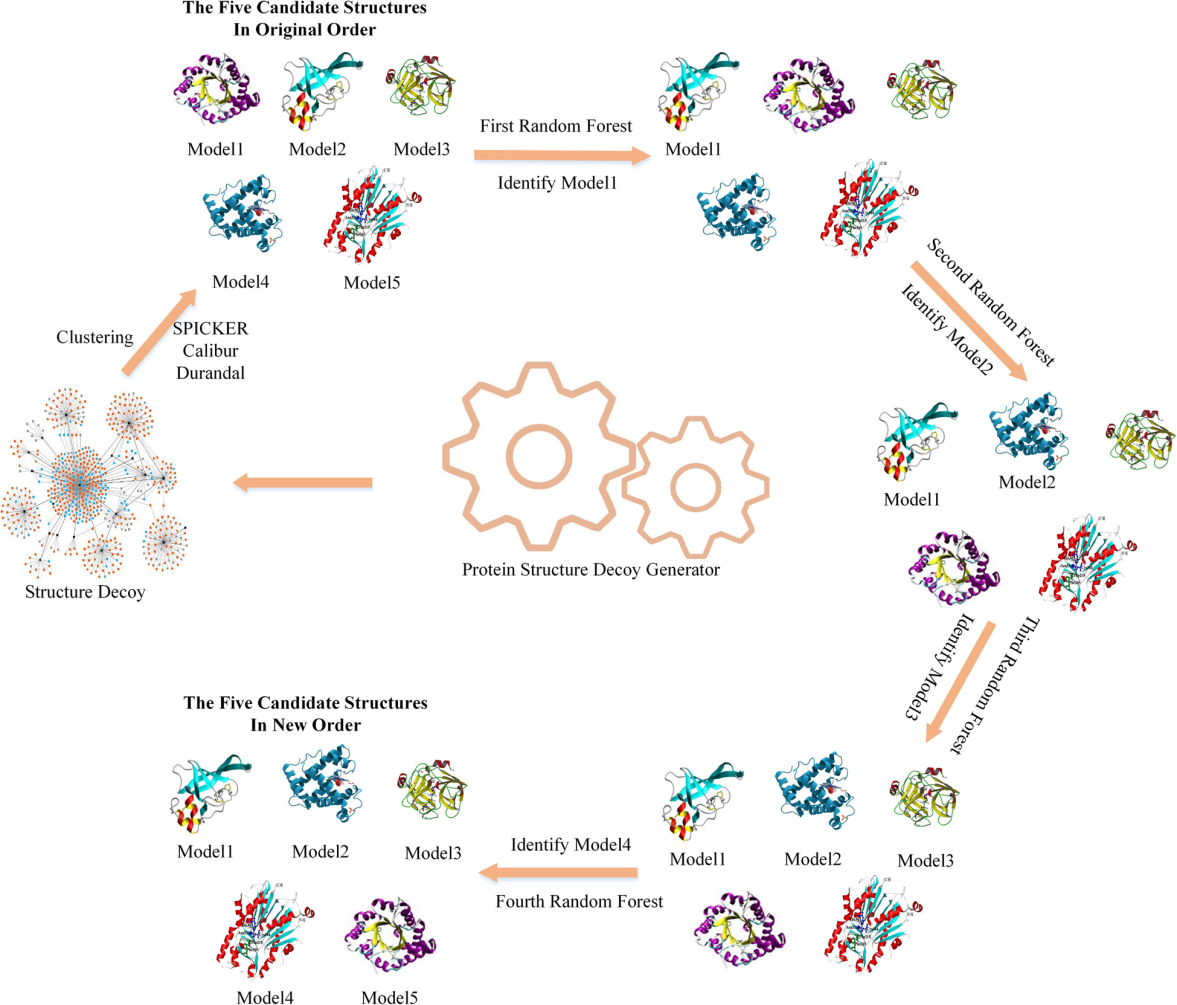
Schematic of the re-ranking method via random forest classification

Random forest classification employs a combination of the bagging algorithm and the random subspace algorithm [13, 14], with a decision tree used as a foundation of the method [15, 16]. Classification accuracy is improved by combining multiple decision trees: *h*_1_(*x*), *h*_2_(*x*), …, *h*_*nTree*_(*x*) [17, 18]. Once the random forest classifier is obtained (Fig. 1), classification of samples of unknown categories is performed. The original data *T* = (*x*_*i*1_, *x*_*i*2_, *x*_*i*3_, …, *x*_*i*6 + *MAXN* × M_, *y*_*i*_), *i* ∈ [1, *N*] (the index i represents ith samples in the original and the index x represents each feature of the random forest.) contains N samples corresponding to 6 + MAXN × *M* features in the dataset. *Y* = *y*_*i*_, *i* ∈ [1, *N*] is the category label that corresponds to the RMSD between each decoy and the native protein structure. y_i_ takes c ≥ 2 values, which represent c classifications. The method used four different random forest to identify the first model, the second model, the third model, the forth model and the fifth model. Each random forest is a binary classification where “1” represents the candidate that has minimum RMSD with native protein and “0” represents the remaining candidates in decoys. We built these four random forest sequentially. After each random forest was completed, we selected candidate that labeled “1” as the best near-native model and removed it from the decoys. At the same time, we used the remaining candidates as the input for the next random forest. The method was done until all candidates were selected. The process of method is shown in Fig. 1.

### Algorithm

The first step involves clustering using each method in order to obtain K clusters [19, 20], followed by ranking by the number of proteins in each category and extracting the top five or three optimal models [21], which are divided into a training set and a test set.

The training set T1 is randomly divided into N sub-datasets which are the number of trees in forest that is set as 100, discretization of each continuous attribute using the dichotomy, and the best classification node is selected from the 6 + MAXN × *M* features using information entropy [22]. The feature with the best value is selected as the best split feature [23], with Eq. (1) showing the calculation method. Until the division of the feature ends, a decision tree is formed, the result is obtained according to the voting criterion. And until the N trees are constructed, the random forest is completed.

1 $$ Entropy(T)=-{\sum}_{i=1}^4{P}_i{\log}_2{P}_i $$

According to Eq. (1), the larger the information entropy, the higher the purity of the data. P_i_ represents the proportion of category i samples relative to the total number of samples. Therefore the training set T1 is divided n parts which equal to the number of attribute values of the feature that is chosen by the information entropy.

Finally, the test set is used to obtain the sorted results [24].

The end conditions of the random forest algorithm are as follows: the decision tree reaches the maximum depth, and the end node impurity reaches the threshold, and the number of samples at the end node reaches the set value, and the features are fully used. The algorithm of random forest is shown in Table 1.

**Table 1:**
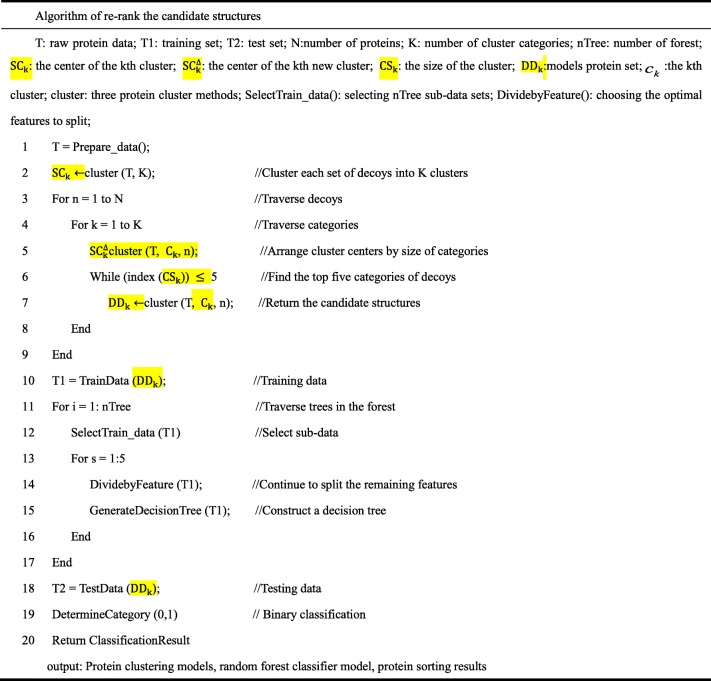
Improved detection of near-native structures via random forest classification

### Evaluation indices

To evaluate the performance of the re-rank method, the RMSD and TM_SCORE are used to evaluate the distance of models to the native structure, respectively.

#### RMSD

As a commonly used measure of the difference between protein structures, RMSD describes variation between two models. The RMSD represents the sample standard deviation of the difference between the predicted value and the observed value. When these differences are estimated by data samples, they are often referred to as residuals, whereas when they are not calculated by samples, the differences are referred to as prediction error. The RMSD is mainly used to aggregate the size of the error in the prediction and often expresses this prediction as a magnitude at different times. The RMSD is a measure of good accuracy and generally used to compare the predicted error of a particular variable between different models [25–27]. RMSD is calculated according to Eq. (2):

2 $$ RMSD=\sqrt{\frac{1}{N}{\sum}_1^N\left({x}_i^2-{x}_j^2\right)+\left({y}_i^2-{y}_j^2\right)+\left({z}_i^2-{z}_j^2\right)} $$

where N is the number of atoms corresponding to the two proteins i and j.

#### TM_SCORE

TM_SCORE measures structural similarity between two protein models. This index addresses global multiple similarity and is insensitive to local structural changes, with the TM_SCORE of random structure pairs generally independent of sequence length. TM_SCORE values are presented as a set (0, 1), where 1 represents a perfect match between two structures. According to calculations of TM_SCORE using structures from the Protein Data Bank, a score > 0.17 corresponds to randomly selected unrelated proteins, whereas a score > 0.5 assumes highly similar folds [28]. TM_SCORE is calculated according to Eq. (3):

3 $$ TM\_ Score=\mathit{\operatorname{Max}}\left[\frac{1}{L_n}{\sum}_i^{L_a}\frac{1}{1+{\left(\frac{d_i}{d_0}\right)}^2}\right] $$

where L_n_ is the sequence length of the native structure, L_a_ is the sequence length of the residue-specific alignment with the template structure, d_i_ is the distance residual between the i^th^ alignment, d_0_ is the scale of the standardized matching difference, and Max indicates the maximum value after optimal spatial superposition.

## Results

### Datasets

Four datasets are employed in the experiments. They are I-TASSER Decoy Set-I, QUARK Decoy Set, CASP10 dataset and CASP11 dataset which are generated by I-TASSER and QUARK (https://zhanglab.ccmb.med.umich.edu/decoys/). These datasets are widely used to evaluate protein decoy clustering [29]. We used I-TASSER Decoy Set-I as a test dataset and the other three datasets as the training sets. Table 2 provides an overview of the four datasets.

**Table 2:** Datasets

Data set	Number of proteins	Average length
I-TASSER Decoy Set-I	43	80
QUARK Decoy Set	145	107
CASP10 dataset	54	212
CASP11 dataset	39	203

The TASSER Decoy Set-I contains a complete set of atomic structure decoys for 56 non-homologous proteins. Among them, 13 proteins whose decoys are not able to cluster into more than five clusters are removed. The remaining 43 proteins are employed in the dataset. The backbone structure was ab initio modeled by I-TASSER, and side-chain atoms were added using Pulchra (http://www.pirx.com/pulchra/index.shtml).

The QUARK Decoy Set contains 145 non-homologous proteins. The backbone structure was ab initio modeled by QUARK, with the all-atom and models of the best candidate generated by ModRefiner (https://zhanglab.ccmb.med.umich.edu/ModRefiner/).

The CASP10 dataset relies upon I-TASSER and QUARK decoys for single-domain proteins in CASP10 that the I-TASSER server predicted as belonging to a single domain. The dataset contains 54 proteins with experimental structures resolved before the CASP10 meeting. The data harbor a gap between the submitted model and the best model among the decoys; therefore, choosing the best model relative to the experimental structure is extremely challenging.

The CASP11 dataset includes decoys generated by I-TASSER and QUARK for CASP11 targets and that the I-TASSER server predicted as belonging to a single domain. Multi-domain targets were ignored to avoid the possibility that ambiguity in domain splitting might render the decoys meaningless. These decoys were used during CASP11.

### Comparison of the three clustering methods with random forest classification

We evaluated the ability of the method to identify near-native structures relative to that of previous methods according to clustering methodology. Predictions were performed across the same time points, with the first false prediction leading to inaccuracies in subsequent predicted models and resulting in poor rankings. The comparative analysis removes the ranked data and ranks the remaining data for subsequent rounds of processing.

#### Comparison of the first model

Because the RMSD between decoy models and the native model is used as a label for the random forest classifier, we assigned model with the lowest RMSD as label “1”, and the remaining models as label “0” to establish a two-category set (0,1) for ranking. However, the percentage of model with “0” is four-fifths and the percentage of model with “1” is one-fifth, there is an imbalance of the training set. We used over-sampling to increase the amount of data in the “1” case, so that we can reduce the imbalance of training set. The 43 sets representing the protein data were submitted for training, with the models having an RMSD of “1” predicted as the first model. Comparing RMSD values between the first model predicted by the random forest classifier and those predicted using the three different clustering methods indicated that our method outperformed the others (Table 3).

**Table 3:** RMSD comparison of the first model of 43 proteins

PDB	Len^a^	Best^b^	SPICKER		Calibur		Durandal	
			Original^c^	RF_SPICKER^d^	Original^c^	RF_Calibur^e^	Original^c^	RF_Durandal^f^
1abv_	103	4.81	13.93	***8.08***	13.17	13.17	13.57	***12.31***
1af7__	72	2.92	5.73	5.73	4.45	4.45	10.28	***3.99***
1ah9_	63	1.88	4.66	4.66	3.31	***2.81***	3.02	3.02
1b4bA	71	4.20	7.18	***5.08***	5.57	5.57	5.54	5.54
1b72A	49	2.36	***4.07***	5.08	***3.23***	3.73	3.23	3.23
1bm8_	99	6.67	7.18	7.18	7.07	7.07	7.48	7.48
1bq9A	53	3.98	7.39	***5.04***	8.18	***6.42***	8.36	8.36
1cewI	108	3.20	3.92	3.92	12.49	***4.28***	3.75	3.75
1cqkA	101	1.40	2.78	2.78	***1.69***	1.95	2.37	2.37
1dcjA_	73	9.31	11.66	***10.45***	12.18	***11.66***	11.97	***9.96***
1di2A_	69	1.32	2.49	2.49	2.62	***2.19***	2.49	2.49
1dtjA_	74	1.58	***2.54***	3.22	2.83	***1.88***	1.88	1.88
1egxA	115	1.93	2.31	2.31	***2.59***	2.95	2.59	2.59
1g1cA	98	2.16	2.97	2.97	2.65	***2.59***	2.49	2.49
1gjxA	77	5.01	7.30	***5.58***	14.09	***13.23***	8.09	8.09
1gnuA	117	4.06	7.09	***6.78***	9.15	***7.74***	9.54	9.54
1gpt_	47	2.79	5.52	5.53	6.29	***4.64***	3.68	3.68
1gyvA	117	2.69	3.78	3.78	***3.41***	3.63	3.39	3.39
1hbkA	89	2.69	3.57	3.57	3.48	***3.52***	3.48	3.48
1itpA	68	4.10	11.23	***8.04***	10.92	***8.07***	11.48	11.48
1jnuA	104	2.30	3.45	3.45	***2.68***	3.21	2.76	2.76
1kjs_	74	4.65	8.67	***5.88***	8.44	***5.89***	8.75	***5.92***
1mkyA3	81	3.68	5.16	5.16	5.54	***5.33***	5.49	5.49
1mla_2	70	2.04	3.18	3.18	2.82	***2.98***	3.38	3.38
1mn8A	84	5.14	6.69	6.69	7.45	7.45	10.38	10.38
1n0uA4	69	3.14	4.59	4.59	4.62	***4.37***	4.28	4.28
1ne3A	56	3.16	***5.12***	6.63	6.09	***4.05***	5.96	5.96
1no5A	93	6.12	10.82	***10.56***	10.69	***10.54***	11	11
1npsA	88	1.81	3.07	3.07	***2.28***	2.74	***6.07***	8.29
1o2fB_	77	4.08	7.41	***7.12***	9.03	***6.80***	3.91	3.91
1ogwA_	77	0.96	1.81	1.81	***1.29***	1.34	2.43	3.00
1pgx_	59	2.79	3.42	3.42	***3.26***	4.19	3.26	3.26
1r69_	61	1.30	2.28	2.28	***1.97***	2.14	1.99	1.99
1shfA	59	1.18	2.86	2.86	***1.49***	2.75	1.29	1.29
1sro_	71	2.59	3.54	***3.00***	***3.54***	3.89	3.54	3.54
1tfi_	47	2.49	***4.61***	5.72	5.08	5.08	4.48	4.48
1thx_	108	1.71	2.67	2.67	***2.26***	2.27	2.10	2.10
1tif_	59	6.47	7.45	7.45	7.57	7.57	***7.62***	9.44
1tig_	88	3.00	9.12	***6.11***	3.58	3.58	4.25	4.25
1vcc_	76	4.52	6.53	6.53	8.13	***6.43***	7.46	7.46
256bA	106	2.75	3.20	3.20	***5.93***	6.23	3.73	***2.78***
2pcy_	99	3.87	5.46	5.46	***1.94***	2.12	4.71	4.71
2a0b_	118	2.05	2.20	2.20	3.48	***2.98***	2.75	2.75
Average	81.09	3.28	5.36	***4.91***	5.53	***4.99***	5.36	***5.15***

^a^:Length of protein sequence

^b^:RMSD between the best model in the decoy and native

^c^:RMSD of the first model predicted by SPICKER,Calibur and Durandal

^d^:RMSD of the first model predicted by the random forest classification from SPICKER results

^e^:RMSD of the first model predicted by the random forest classification from Calibur results

^f^:RMSD of the first model predicted by the random forest classification from Durandal result

The RMSD in bold and italic indicates RF(RF_SPICKER, RF_Calibur and RF_Durandal) methods obtain lower RMSD than their original methods

Use of the random forest classifier ranked the candidate structures with higher accuracy according to average RMSD. Twelve of the models predicted by the random forest classifier were closer to the native structure than those predicted by SPICKER, 27 were the same, and four were inferior. The average RMSD decreased 8.40% from 5.36 to 4.91 after ranked by random forest classifier. Twenty-one of the models predicted by the random forest classifier were closer to the native structure than those predicted by Calibur, eight were the same, and 14 were inferior. Finally, six of the models predicted by the random forest classifier were closer to the native structure than those predicted by Durandal, 35 were the same, and two were inferior. These data indicated that the random forest classifier allowed more accurate order of candidate structures exhibiting the highest degree of similarity to the native structure relative to the three other methods.

#### Comparison of the second model

**Fig. 2:**
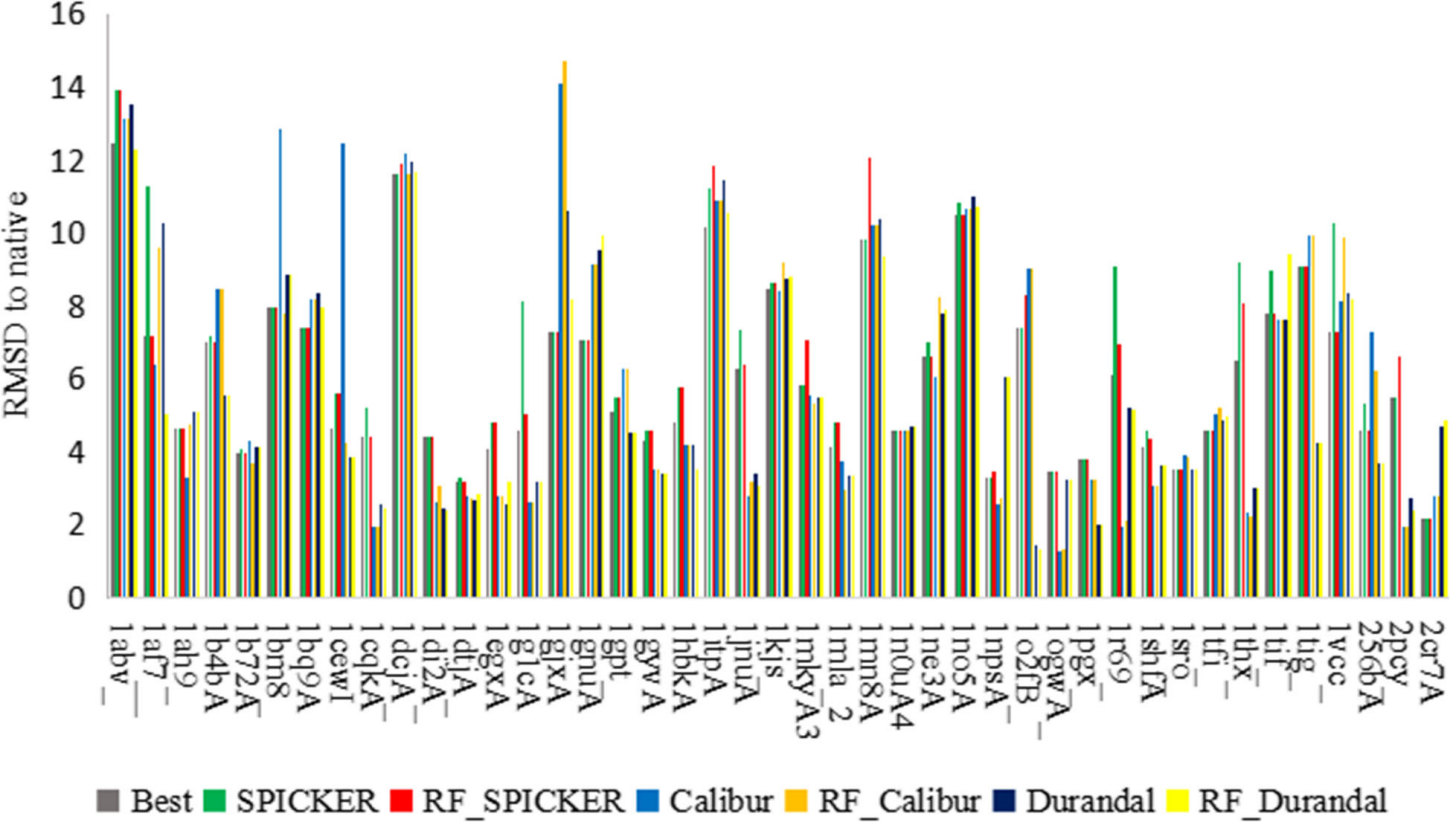
Comparison of RMSD of the second model in the absence of the first model

After removal of the first model from the dataset, we followed the same algorithmic procedure to establish the optimal RMSD values between decoy models and the native structure, resulting in another two-category set (0,1). However, the percentage of model with “0” is three-fourths and the percentage of model with “1” is one-fourth. We used over-sampling to overcome the imbalance of training set. Comparing RMSD values between the first model predicted by the random forest classifier and those predicted using the three different clustering methods indicated that our method outperformed the others (Fig. 2).

Use of the random forest classifier generated predictions with higher accuracy according to average RMSD. Fifteen of the models predicted by the random forest classifier were closer to the native structure than those predicted by SPICKER, 22 were the same, and six were with higher RMSDs, resulting in a 21% increase in accuracy. Eleven of the models predicted by the random forest classifier were closer to the native structure than those predicted by Calibur, 19 were the same, and 13 were worse, resulting in a 4% increase in accuracy. Sixteen of the models predicted by the random forest classifier were closer to the native structure than those predicted by Durandal, 19 were the same, and eight were worse, resulting in a 18% increase in accuracy. These data indicated that the random forest classifier allowed more accurate prediction of models exhibiting the highest degree of similarity to the native structure relative to the three other methods.

#### Comparison of the third model and the fourth model

**Fig. 3:**
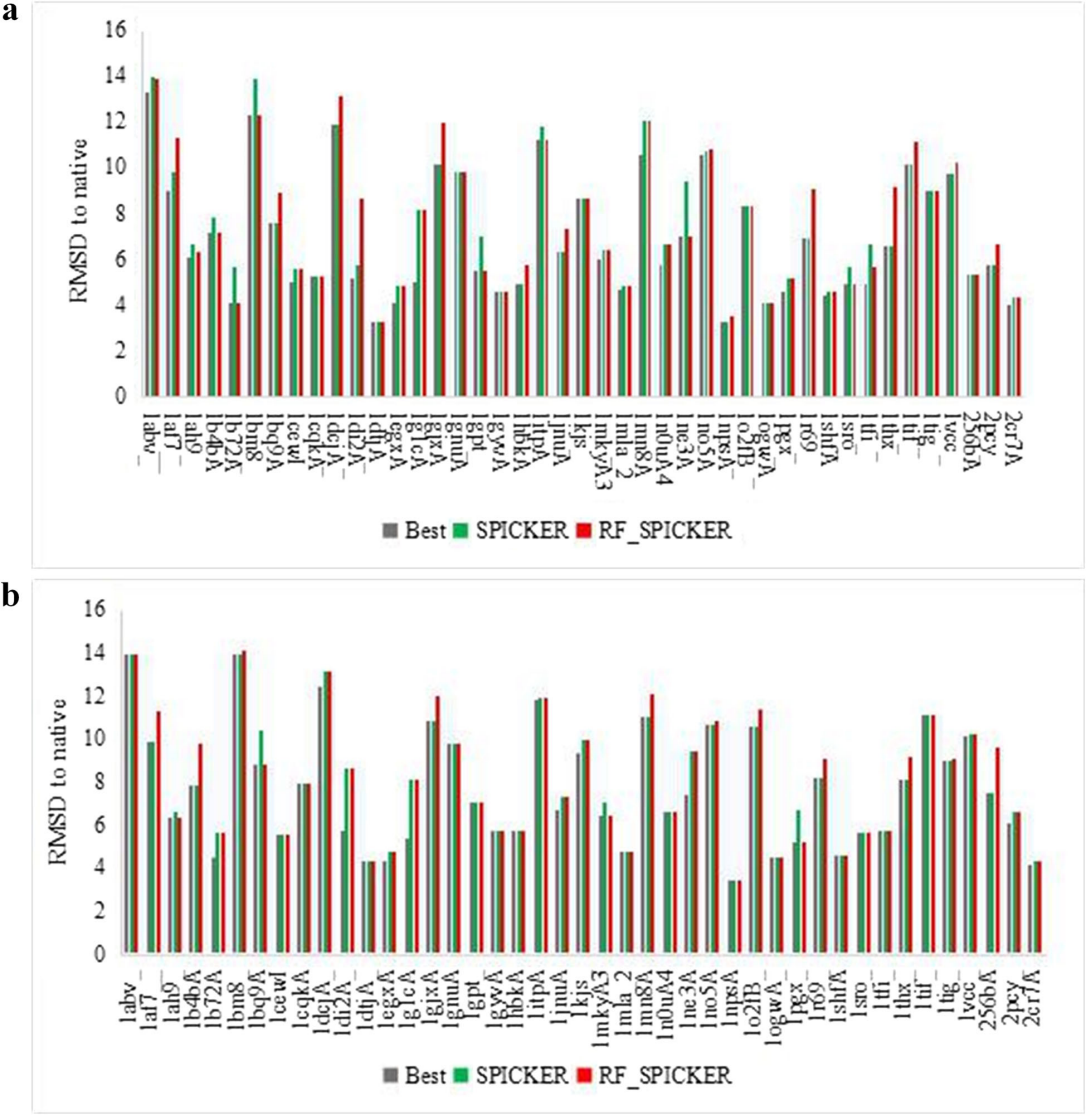
Comparison of the RMSD of the third model and the fourth model. **a**. Comparison of the RMSD of the third model. **b**. Comparison of the RMSD of the fourth model

Since Calibur and Durandal usually predict only the three of the near-native candidate structures, while SPICKER can predict five structures, the comparisons of the third and the fourth models are only implemented against SPICKER. Comparing RMSD values between the third and the fourth model predicted by the random forest classifier and those predicted using the three different clustering methods indicated that our method outperformed the others (Fig. 3). In the Fig. 3a, the random forest classifier ordered predictions with higher accuracy according to average RMSD. Sixteen of the models predicted by the random forest classifier were closer to the native structure than those predicted by SPICKER, 17 were the same, and ten were worse, resulting in a 14% increase in accuracy. In the Fig. 3a, Use of the random forest classifier generated predictions with higher accuracy according to average RMSD. Eleven of the models predicted by the random forest classifier were closer to the native structure than those predicted by SPICKER, 27 were the same, and five were worse, resulting in a 14% increase in accuracy. These data indicated that the random forest classifier allowed more accurate prediction of models exhibiting the highest degree of similarity to the native structure relative to SPICKER.

### Comparison of the numbers of correct predictions

**Fig. 4:**
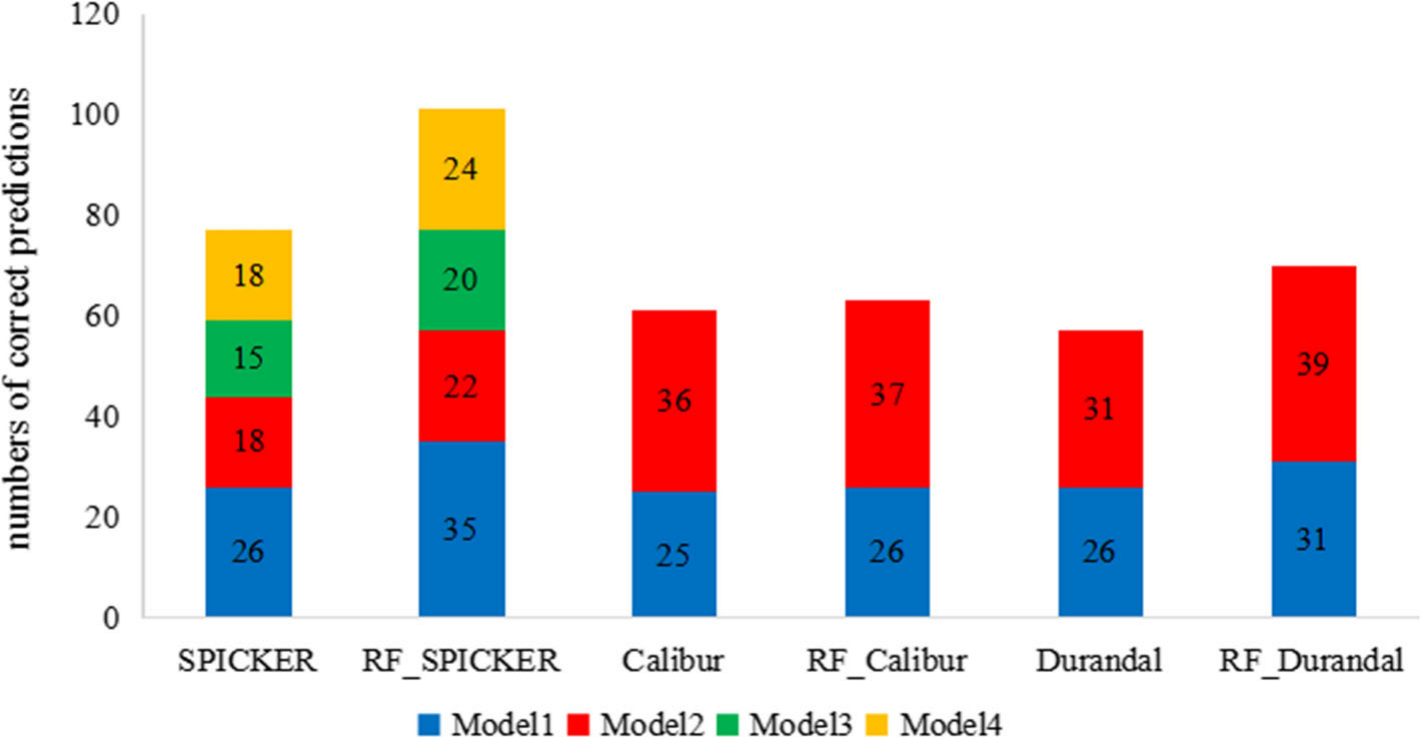
Comparison of the numbers of correct predictions

The Fig. 4 indicated that the random forest classifier allowed more accurate prediction of models exhibiting the highest degree of similarity to the native structure relative to three clustering methods. After re-ordered by RF_SPICKER, 35(81.39%) out of 43 first models are exactly identified, while SPICKER only correctly identified 26(60.46%) first models. When detecting the second third and fourth models, RF_SPICKER correctly predicted 4, 5 and 6 targets more than SPICKER, respectively. Even if Calibur and Durandal usually predict only three near-native candidate structures, RF_Calibur and RF_ Durandal successful predicted 1 and 5 more targets than Calibur and Durandal on the first model respectively. And they successful predicted 1 and 8 more targets on the second model respectively.

## Discussion

1dcj is a small protein encoded by the yhhP gene in the *Escherichia coli* database. Its high precision NMR (Nuclear Magnetic Resonance) structure is identified by Katoh E and his colleagues at 2000 [30–32]. In the previous research the cell division process is related to 1dcj although the precise biological function of this protein has not been yet identified. The serum glycoprotein C5a(1kjs) is derived from the proteolytic cleavage of complement protein C5, has been implicated in the pathogenesis of a number of inflammatory and allergic conditions [16, 33]. The three-dimensional structure is detected by two-dimensional NMR. The computational structures are very useful for protein functional and evolutional understanding.

**Fig. 5:**
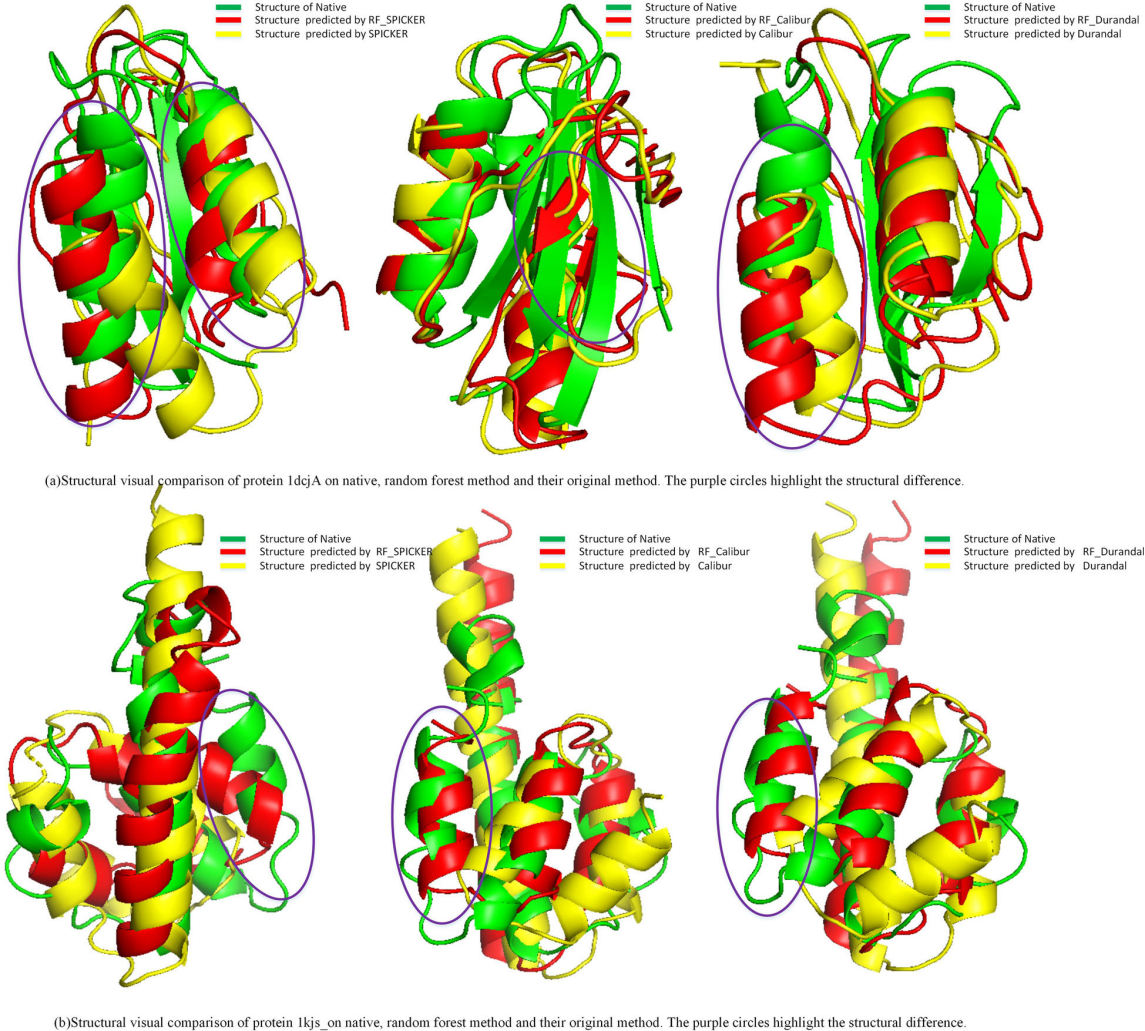
Visual Comparison of random forest classifier and current prediction methods on 1dcjA and 1kjs_

Visual structural comparisons of native, SPICKER, Calibur and Durandal are shown in the Fig. 5a and b. The native structure is in green, the first models detected by SPICKER, Calibur and Durandal are in yellow, and the re-ranked models predicted via random forest classification are in red. In the visual comparison on 1dcj, both SPICKER model (1dcj, RMSD 11.66) and RF_SPICKER model (1dcj, RMSD 10.45) successful built two helixes in the purple circles, but the helixes of RF_SPICKER model are more closer to the native structure. The native structure of 1dcj has three beta-strand motifs. Although prediction of the three-dimensional structure of beta-strand is commonly regarded as difficult task, the random forest classification successfully choose RF_Calibur model (1dcj, RMSD 11.66) with one beta-strand as the first model. Unfortunately Calibur choose the model (1dcj, RMSD 12.18) without any beta-strand. The main difference between Durandal model (1dcj, RMSD 11.95) and RF_Durandal model (1dcj, RMSD 9.96) is the location of first helix region. On the protein 1kjs, SPICKER model (1kjs, RMSD 8.67) completely failed to build the right-side short helix, while the RF_SPICKER model (1kjs, RMSD 5.88) has this short helix and only the direction of the helix is not exactly consistent with the native helix. In Calibur and Durandal model comparison, RF_Calibur model (1kjs, RMSD 5.89) and RF_Durandal model (1kjs, RMSD 5.92) successfully built the short helix rather than Calibur model (1kjs, RMSD 8.44) and Durandal model (1kjs, RMSD 8.74) and well aligned with the native model.

## Conclusion

This study presented a method re-order the candidate near-native structures by random forest classification after the clustering methods explored the five or three candidate structures. The method employed four binary classifier to detect the first, second, third, fourth and fifth model with protein features, inter-cluster features and intra-cluster features. To evaluate the performance of the method four widely-used datasets, I-TASSER Decoy Set-I, QUARK Decoy Set, CASP10 dataset and CASP11 dataset, are employed. Comparison with three dominated methods, the method decreased the average RMSD 8.40% from 5.35 to 4.91 for SPICKER, decreased 9.76% from 5.53 to 4.99 for Calibur and decreased the average RMSD 3.91% from 5.36 to 5.15 for Durandal on the first model.

## Data Availability

The extracted data supporting the conclusions of this article is included within the article. Dataset can be access from https://zhanglab.ccmb.med.umich.edu/decoys/

## Abbreviations

3D: Three-dimensional
CASP: Computer automated stowage planning
NMR: Nuclear magnetic resonance
PSSM: Position-specific scoring matrix
RF_Calibur: RMSD of model predicted by the random forest classification from Calibur results
RF_Durandal: RMSD of model predicted by the random forest classification from Durandal results
RF_SPICKER: RMSD of model predicted by the random forest classification from SPICKER results
RMSD: Root mean squared error
TM_SCORE: Template modeling score
